# Epilepsy‐heart syndrome: Back to the future!

**DOI:** 10.1111/epi.18597

**Published:** 2025-08-05

**Authors:** Guilherme L. Fialho, Katia Lin

**Affiliations:** ^1^ Cardiology Division Federal University of Santa Catarina Florianopolis Brazil; ^2^ Medical Sciences Postgraduate Program Federal University of Santa Catarina Florianopolis Brazil; ^3^ Neurology Division Federal University of Santa Catarina Florianopolis Brazil; ^4^ Center for Applied Neurosciences Federal University of Santa Catarina Florianopolis Brazil

To the Editors,

We read with great interest the article titled “Epilepsy‐Heart Syndrome: Concept, Clinical Context, and Opportunity for Integrated Care” by Mbizvo et al.^1^ In their work, the authors propose a bidirectional relationship between epilepsy and cardiac health. On one hand, cardiac diseases such as arrhythmias, hypertension, hyperlipidemia, and heart failure can lead to impaired cerebral perfusion and altered neurological excitability, and on the other, seizures could lead to cardiac catecholaminergic toxicity and/or ischemia and autonomic dysfunction leading to myocardial injury. They highlight the importance of integrated care between neurologists and cardiologists to face some challenges such as ictal asystole and others.

Medical knowledge has dramatically improved over the past few centuries. This led to the emergence of numerous specialties from the broad discipline of internal medicine and, later, to subspecialties within each specialty.^2^ This specialization mirrored the remarkable progress in diagnostics, therapeutics, and medical technology, and as a result of this evolution, the clinical outcomes of uncountable conditions such as acute myocardial infarction, heart failure, and valvular heart disease have improved significantly.

The neural and cardiovascular systems are intricately interconnected, working together to maintain physiological homeostasis. However, in pathological conditions, this bidirectional relationship can be disrupted through three main mechanisms. First, neurological insults can precipitate cardiac dysfunction, as observed in neurogenic heart disease and stress‐induced cardiomyopathy. Second, primary cardiac conditions may have neurological consequences, as seen in cardioembolic strokes or cognitive decline associated with atrial fibrillation and heart failure. And third, shared risk factors—such as aging, hypertension, and diabetes—or specific disorders like neurocardiogenic syncope and genetic channelopathies can concurrently affect both systems.^3^, ^4^, ^5^

Altered brain activity could be the intersection between epilepsy and cardiovascular disease. There is increasing evidence of a bidirectional relationship between metabolic syndrome and amygdalar dysfunction^6^, ^7^ through neurobiological, neuroendocrine, and metabolic pathways. Metabolic syndrome, characterized by central obesity, insulin resistance, hypertension, and dyslipidemia, is associated with neuroinflammation, synaptic dysfunction, and altered neurotransmitter balance, all of which can impact amygdalar function and connectivity with other brain regions.^6^ Additionally, amygdalar activity is linked to the progression of visceral adiposity and inflammation.^7^

Amygdala involvement in individuals with epilepsy is linked to altered heart rate variability and heightened risk for sudden unexpected death in epilepsy.^8^, ^9^, ^10^ Amygdalar function has been further linked to reduced left ventricular ejection fraction, perfusion, and an increased risk of takotsubo cardiomyopathy.^11^, ^12^ Tawakol and colleagues showed that, among 293 individuals followed for 3.7 years, increased amygdalar activity was associated with increased bone marrow activity, arterial wall inflammation, and a hazard ratio (HR) of 1.59 for cardiovascular events.^13^

Another important link between cardiovascular and neurological health is cardiovascular fitness. Nyberg et al. analyzed more than 1 million individuals for up to 40 years and found that low and medium cardiovascular fitness at age 18 years increased the risk of future epilepsy by up to 79% (HR = 1.79, 95% confidence interval [CI] = 1.57–2.03) and 36% (HR = 1.36, 95% CI = 1.27–1.45), respectively, compared to high cardiovascular fitness.^14^ The impact of cardiovascular fitness on cardiovascular disease, cancer, and cardiovascular and all‐cause mortality is well known.^15^, ^16^

The “epilepsy‐heart syndrome” proposed by Mbizvo et al.^1^ builds upon the previous “neuro‐cardiovascular continuum” proposed by Fialho et al. in 2019^4^ and the “epileptic heart” proposed by Verrier et al. in 2020^17^ and takes us back to a more integrative approach to our patients, acknowledging that cooperation between neurologists and cardiologists is the future of optimal care.

## AUTHOR CONTRIBUTIONS

Guilherme L. Fialho and Katia Lin conceptualized, designed, wrote, and revised this letter equally.

## CONFLICT OF INTEREST STATEMENT

K.L. holds a National Council for Scientific and Technological Development–CNPq PQ1C Research Fellowship (process number 306916/2023‐1). G.L.F. has no conflicts of interest. We declare that this letter describes original work and there is no redundant or duplicate publication related to this letter. Also, this submission is not under review at any other publication. We take full responsibility for the data, the analyses and interpretation, and the conduct of the research; we have full access to all of the data; and have the right to publish any and all data separate and apart from any sponsor. We declare that all authors and contributors have seen and agreed with the contents of this letter, the International Committee of Medical Journal Editors requirements for authorship have been met, and both author believe that the letter represents honest work. We declare that there is no figure or video of any recognizable participant in this letter. We confirm that we have read the Journal's position on issues involved in ethical publication and affirm that this report is consistent with those guidelines.

## Data Availability

All data supporting the findings of this letter are available from the corresponding author upon request.
